# X-ray Phase Contrast Tomography Serves Preclinical Investigation of Neurodegenerative Diseases

**DOI:** 10.3389/fnins.2020.584161

**Published:** 2020-11-09

**Authors:** Francesca Palermo, Nicola Pieroni, Laura Maugeri, Ginevra Begani Provinciali, Alessia Sanna, Inna Bukreeva, Lorenzo Massimi, Maura Catalano, Margie P. Olbinado, Michela Fratini, Antonio Uccelli, Giuseppe Gigli, Nicole Kerlero de Rosbo, Claudia Balducci, Alessia Cedola

**Affiliations:** ^1^TomaLab, Institute of Nanotechnology, CNR, Rome, Italy; ^2^Dipartimento di Fisica, Università della Calabria, Rende, Italy; ^3^Dipartimento di Morfogenesi e Ingegneria Tissutale, Sapienza Università di Roma, Rome, Italy; ^4^Swiss Light Source, Paul Scherrer Institut X-ray Tomography Group, Villigen, Switzerland; ^5^Department of Neurosciences, Rehabilitation, Ophthalmology and Maternal-Fetal Medicine (DINOGMI), University of Genoa, Genoa, Italy; ^6^Ospedale Policlinico San Martino IRCCS, Genoa, Italy; ^7^Institute of Nanotechnology, CNR, Università del Salento, Lecce, Italy; ^8^Istituto di Ricerche Farmacologiche Mario Negri IRCCS, Milan, Italy

**Keywords:** X-ray phase contrast tomography, preclinical disease models, Alzheimer’s disease, multiple sclerosis, 3D imaging

## Abstract

We report a qualitative study on central nervous system (CNS) damage that demonstrates the ability of X-ray phase contrast tomography (XPCT) to confirm data obtained with standard 2D methodology and permits the description of additional features that are not detected with 2D or other 3D techniques. In contrast to magnetic resonance or computed tomography, XPCT makes possible the high-resolution 3D imaging of soft tissues classically considered “invisible” to X-rays without the use of additional contrast agents, or without the need for intense processing of the tissue required by 2D techniques. Most importantly for studies of CNS diseases, XPCT enables a concomitant multi-scale 3D biomedical imaging of neuronal and vascular networks ranging from cells through to the CNS as a whole. In the last years, we have used XPCT to investigate neurodegenerative diseases, such as Alzheimer’s disease (AD) and multiple sclerosis (MS), to shed light on brain damage and extend the observations obtained with standard techniques. Here, we show the cutting-edge ability of XPCT to highlight in 3D, concomitantly, vascular occlusions and damages, close associations between plaques and damaged vessels, as well as dramatic changes induced at neuropathological level by treatment in AD mice. We corroborate data on the well-known blood-brain barrier dysfunction in the animal model of MS, experimental autoimmune encephalomyelitis, and further show its extent throughout the CNS axis and at the level of the single vessel/capillary.

## Introduction

Neurodegeneration is a process by which a progressive loss of neuronal structure and function occurs in many central nervous system (CNS) pathologies; it is generally associated with neuroinflammation. Neurodegenerative diseases are presently incurable and current therapies have minimal or no significant effect in reversing the CNS damage.

The research in human neurological diseases has greatly benefited from pre-clinical research in experimental *in vitro* and *in vivo* models. In this context, for example, several of the therapeutic approaches that have led in the past 30 years to an increasing number of drugs for multiple sclerosis (MS), such as glatiramer acetate (Ben-Nun et al., 1996) and natalizumab (Yednock et al., 1992) in particular, have been developed in its murine model, experimental autoimmune encephalomyelitis (EAE) (Constantinescu et al., 2011).

However, new efforts are necessary for the comprehension of disease mechanisms and monitoring of therapeutic approaches. In particular, the research in neurodegenerative diseases requires tools enabling the visualization of disease-relevant networks, such as the vascular and neuronal networks (VN and NN), and of affected cells, as well as the monitoring of treatment efficacy. The techniques currently used to investigate damage in the VN and NN at cellular level suffer from several limitations. In particular, 2D imaging (immuno-histochemistry and electron microscopy) restricts spatial coverage, entails destructive sample preparation, and is only applicable at the *ex vivo* level. Although jumping from 2D to 3D represented an outstanding breakthrough in the general quality of imaging and information obtained, magnetic resonance imaging (MRI), positron emission tomography (PET), and X-ray-computed tomography fail to provide a satisfactory answer to the unmet medico-imaging needs for these diseases. Thus, MRI and PET are limited in terms of spatial resolution, and X-ray computed tomography, whilst providing 3D visualization of X-ray-absorbing tissues, fails in the analysis of soft tissues, such as the CNS.

These severe limitations in 3D imaging can be overcome by the more advanced X-ray phase contrast tomography (XPCT), which provides much higher resolution and contrast at cellular level also in soft tissues. XPCT makes possible the simultaneous multi-scale 3D biomedical imaging of neuronal and vascular networks, ranging from cells through to brain as a whole. XPCT revolutionizes X-ray imaging and removes its main limitation of poor image contrast arising from low attenuation differences. XPCT increases the contrast of all details and enables the detection of features classically considered as “X-ray invisible.”

A key ability of XPCT is the possibility to generate a 3D *multiscale* image of the whole brain, which displays information on the NN and VN *simultaneously* (Fratini et al., 2015; Bukreeva et al., 2017; Begani Provinciali et al., 2020). In previous works (Cedola et al., 2017; Massimi et al., 2019, 2020), we exploited this unique feature of XPCT to evaluate morphological alterations in the VN and NN, both in EAE and in the APP/PS1dE9 mouse model of Alzheimer’s disease (AD).

In this work, we move forward in these studies toward a deeper understanding of *specific* hot issues in MS and AD. Namely, in EAE, effective 3D imaging has allowed us to confirm disease-related alterations in blood-brain barrier (BBB) permeability, demonstrating its spreading throughout the affected tissue. In the APP/PS1 mouse model, we have investigated the effect of a new therapeutic approach on β–amyloid (Aβ) plaques, the main neuropathological hallmark of AD (Jack et al., 2010).

## Materials and Methods

### Sample Preparation

Experimental autoimmune encephalomyelitis sample: An eight-week-old C57Bl/6J female mouse, weighing 18.5 g, purchased from Harlan Italy, was immunized as described before (Mendel et al., 1995) by subcutaneous injection (200 μl total) at two sites in the flank with an emulsion of 200 μg myelin oligodendrocyte glycoprotein (MOG) peptide 35–55 (Espikem) in incomplete Freund adjuvant (Difco) containing 600 μg Mycobacterium tuberculosis (strain H37Ra; Difco). The mouse was injected (100 μl total) in the tail vein with 400 ng pertussis toxin (Sigma-Aldrich) immediately and 48 h after immunization. The mouse was scored daily for clinical manifestations of EAE on a scale of 0–5 (Mendel et al., 1995), and sacrificed by CO2 inhalation at onset of clinical manifestations (day 11 after immunization), with a clinical score of 3.5. The brain and spinal cord were dissected out, with the spinal cord being divided into 3 parts, cervical (C1–C7), thoracic (T1–T13, and lumbar/sacrococcygeal (L1–S4). The tissues were fixed in 4% paraformaldehyde for 24 h, then stored in 70% ethanol until XPCT.

All animals are housed in pathogen-free conditions and treated according to the Italian and European guidelines (Decreto Legislativo 4 marzo 2014, n. 26, legislative transposition of Directive 2010/63/EU of the European Parliament and of the Council of 22 September 2010 on the protection of animals used for scientific purposes), with food and water *ad libitum*. The research protocol was approved by the Ethical Committee for Animal Experimentation of the University of Genoa (Prot. 319).

AD samples: APPswe/PS1dE9 transgenic male mice [B6C3 – Tg(APPswe, PSEN1dE9)85Dbo/Mmjax mice], which express a chimeric mouse/human amyloid precursor protein (Mo/HuAPP695swe) and a mutant human presenilin 1 (PS1-dE9), were purchased from Jackson Laboratories (United States). They are referred to thereafter as AD mice.

All animals were housed in a SPF facility in groups of 4 in standard mouse cages containing sawdust with food (2018S Envigo diet) and water *ad libitum*, under conventional laboratory conditions (room temperature: 20 ± 2°C; humidity: 60%) and a 12/12 h light/dark cycle. The IRFMN adheres to the principles set out in the following laws, regulation, and policies governing the Care and Use of Laboratory Animals: Italian Governing Law (D.lgs 26/2014; Authorization n. 19/2008-A issued March 6, 2008 by Ministry of Health); Mario Negri Institutional Regulations and Policies providing internal authorization for persons conducting animal experiments (Quality Management System Certificate – UNI EN ISO 9001:2015 – Reg. N° 6121); the NIH Guide for the Care and Use of Laboratory Animals (2011 edition) and EU directives and guidelines (EEC Council Directive 2010/63/UE). The statement of Compliance (Assurance) with the Public Health Service (PHS) Policy on Human Care and Use of Laboratory Animals has been reviewed (9/9/2014; Animal Welfare Assurance #A5023-01). The mice were used for the experiment at 18 months of age. One AD mouse was treated intranasally (25 μl total volume for both nostrils) once weekly for eight consecutive weeks with the concentrated secretome of mesenchymal stem cells (MSC), that is the conditioned supernatant of cultured MSC (MSC-CS), as described previously (Santamaria et al., 2020); the other untreated mouse received concentrated medium intranasally according to the same regimen. Mice were killed under CO_2_ inhalation. For micro-XPCT, the brains were removed and post-fixed in 4% paraformaldehyde overnight and subsequently stored in 70% ethanol at 4°C until XPCT. For holo-nano-XPCT and nano-XPCT, the brains were removed and specific regions (frontal cortex, hippocampus) were cut and placed in 0.1 M cacodylate buffer pH 7.2, containing 2.5% glutaraldehyde for 3 h at room temperature. Brain samples were postfixed in osmium tetroxide (1% in 0.1 M cacodylate buffer, pH 7.2; 1 h) and uranyl acetate (1% in water; 1 h). Samples were then dehydrated through a graded ethanol series (70/95/100%), put in propylene oxide, and embedded in resin (Poly-Bed; Polysciences, Inc., Warrington, PA, United States) at 42°C overnight and for 2 days at 60°C. The blocks were kept at 4°C.

### Micro-XPCT

The XPCT experiments were performed at the ID17 beamline of the European Synchrotron Radiation Facility (ESRF, Grenoble, France) and at the ANATOMIX beamline of Synchrotron SOLEIL (Paris, France), in free-space propagation mode (Bravin et al., 2012).

Data acquisition at ID17 was performed using monochromatic incident X-ray energy of 35 keV. The sample-detector distance was set at 2.3 m. The detector has an effective pixel size of 3.05 μm. The tomography was produced by means of 2000 projections covering a total angle range of 180°. The acquisition time for each angular position was 300 ms. The total sample volume was acquired in about 15 min. Data pre-processing, phase retrieval, and tomographic reconstruction were performed with SYRMEP Tomo Project software (Brun et al., 2017; Massimi et al., 2018) and optimized scripts.

The experiment at ANATOMIX beamline was carried out with a filtered white beam peaked around 20 keV. The propagation distance was 0.2 m. The effective pixel size was 3.25, resulting from 2× optics coupled with Orca Flash 4.0 camera (sensor type CMOS, sensor array size 2048 × 2048, pixel size 6.5 μm 16-bit nominal dynamic range). The experiment was performed acquiring 4000 projections in 360° scan mode. 360° scan mode or extended field of view (FOV) mode (half-acquisition mode) pertain specifically to the acquisition of sample horizontally larger than the FOV of the camera. In this acquisition mode, the sample is positioned outside the rotation center of the stage so that half of the sample is outside the FOV. We acquired over an angular range of 360°. The projections acquired during the first 180° provide information on the first part of the sample, while the projections acquired from 180 to 360° provide information on the sample region which was initially out of the FOV. Data pre-processing, phase retrieval, and tomographic reconstruction were performed with PyHST software package. The tomography color scale is a gray-shade scale from black to white. Least dense tissues appear black, structures with highest density appear white.

### Holographic Nano-XPCT

The holographic (holo-)nano-XPCT experiment was carried out at Nano-Imaging ID16A beamline of the ESRF. A pair of multilayer-coated Kirkpatrick-Baez optics was used to focus the X-rays (∼30 nm) at 17 keV. The sample is put in the divergent beam downstream of the focus to produce magnified phase contrast images. The projection geometry also allows zooming into specific regions of a large sample by combining scans with different magnifications and FOV (Mokso et al., 2007; Bartels et al., 2015). By measuring the Fresnel diffraction patterns at different effective propagation distances, the phase maps of the sample can be retrieved via holographic reconstruction, this so-called phase-retrieval procedure (Cloetens et al., 1999) being implemented using GNU Octave software. Magnified radiographs were recorded onto an X-ray detector using a FReLoN-charged coupled device. For one tomography scan, 1500 projections were acquired with 0.32 s exposure time and 50 nm effective pixel size. Tomography scans at four different foci-to-sample distances were acquired to complete one holotomography scan. The tomographic reconstruction was obtained with ESRF PyHST software package. In this kind of images, the shades of gray are proportional to electron density, with black corresponding to the highest value of the density spectrum, whereas white corresponds to the lowest value, hence to features of lowest density.

### Nano-XPCT

The nano-XPCT experiment was performed at TOMCAT beamline of the Swiss Light Source at Paul Scherrer Institut (Villigen, Switzerland). An incident monochromatic x-ray beam with an energy of 17 keV was used. In order to obtain an effective pixel size of 325 nm, a 20× lens was coupled with a pco.edge 5.5 camera (sCMOS-technology, 2560 × 2160 pixels, 6.5 μm pixel size and a 16-bit nominal dynamic range). The experiment was performed in free-space propagation mode, with a single propagation distance of 50 mm. For one tomography scan, 1500 projections were acquired with 0.12 s exposure time. Data reconstruction was obtained with TOMCAT plug-in of ImageJ software. As for micro-XPCT, the gray scale is proportional to the electron density of the tissues.

### Image Analysis

We removed the ring artifacts by improved frequency filtering (Massimi et al., 2018). Image analysis was performed using ImageJ software^1^. To enhance the contrast, we used *z*-projection of maximum and minimum intensity. *Z*-Projection consists in projecting an image stack along the axis perpendicular to the image plane (the so-called “*z*” axis). Performing the projection of the maximum (or minimum) intensity creates an output image of which each pixel contains the maximum (or minimum) value over all images in the stack at the particular pixel location. On the one hand, maximum intensity projection (MAX) enlightens high density structures such as neurons and amyloid plaques. On the other hand, minimum intensity projection (MIN) emphasizes low-density details such as the lumen of the vessels. To simultaneously visualize features with low and high density, images obtained from MAX and MIN were added together.

XPCT provides intrinsic 3D information of the samples measured. Three-dimensional spatial distribution of anatomical structures can be effectively rendered using VG studio Max software. To isolate and enlighten particular structures, image segmentation is required. This procedure groups features with the same density by selecting the corresponding portion of the gray-level-histogram intensity. The segmentation and 3D rendering, performed with VGstudioMax, can be exploited to simultaneously visualize different structures, furthering the study of the interaction among biological networks.

## Results

### XPCT for Brain Imaging

The ambitious objective to explore the CNS from the whole organ down to the single cell through a detailed 3D imaging, whilst preserving tissue chemistry and structure, can nowadays be achieved through XPCT. Its most compelling achievement is the possibility to investigate the highly complex VN and NN in the context of the tissue as a whole and, therefore, compare physiological vs. pathological states at the level of crucial disease targets.

**FIGURE 1:**
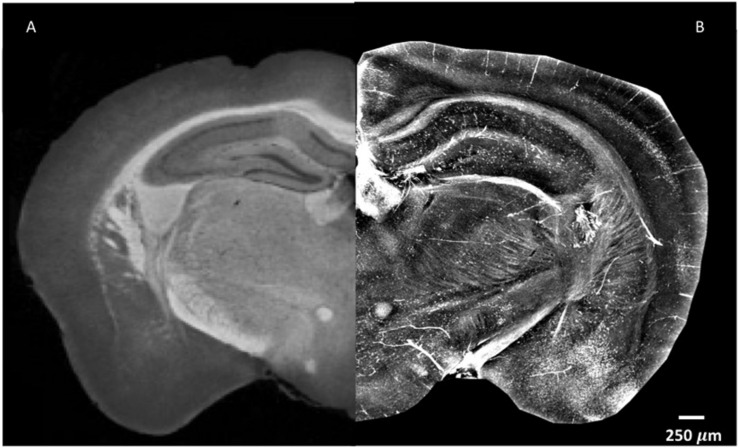
XPCT is a powerful technique that provides high-contrast resolution without requirement for a contrast agent. The image shown puts together parts of naïve mouse brain with the left side assessed by standard X-ray micro-tomography with a mixture of 1% iodine and 90% methanol as contrast agent **(A)** as reported in Zikmund et al. (2018) JINST 13 C02039, while the right side was generated by XPCT **(B)**. Small variations in density appear much more evident in XPCT imaging. Both images were obtained as *z*-projection of maximum intensity over 300 μm.

In order to explore the complexity of the tissue, standard X-ray tomography of the brain requires the application of contrast agents, as shown in Figure 1A. As can be seen, the difference in contrast between the cortex and the thalamus is clear, and the corpus callosum and the mammillothalamic tract are well defined. However, despite the use of contrast agent, little information can be extracted on nerve cells in the cortex and on nerve fibers (Figure 1A). Contrary to standard X-ray tomography, the contrast in the XPCT images is not proportional to the X-ray attenuation inside the sample but is proportional to the refraction of the X-ray beam crossing the sample. Since in the biomedical samples the refraction effect can be 1000 times higher with respect to the absorption effect, XPCT is a unique technique of tomography to image low-absorbing tissues. Figure 1B shows the same anatomic district imaged in Figure 1A, using XPCT, but the richness of details in this case is quite impressive with respect to Figure 1A. Even though a contrast agent was not used (see below), the vascularization in the cortex clearly appears, and the cells and fibers are imaged with optimal contrast.

**FIGURE 2:**
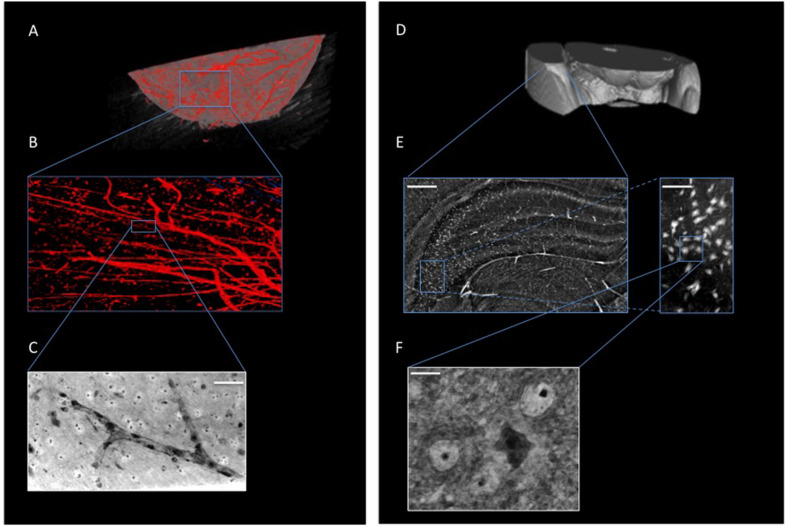
XPCT is a multiscale 3D technique that enables the simultaneous visualization of different anatomical structures within biological tissues without sectioning or staining, and allows the imaging from whole-organ level down to nanometric details. **(A,B)** 3D-rendering of naïve mouse brain vasculature (in red). **(C)** Holo-nano-XPCT image of a single capillary (acquired at ID16, ESRF; scale bar, 50 μm). **(D–F)** XPCT images showing the multiscale approach to analyze the neuronal network down to the single cell; **(E)** Inset of **(D)** showing a micro-XPCT detail of brain in the hippocampal region (scale bar, 250 μm) and a magnification (scale bar, 80 μm) demonstrating the ability of micro-XPCT to go down to cell level; micro-XPCT measures were acquired at ID17, ESRF. **(F)** Holo-nano-XPCT image highlighting the ability of XPCT to distinguish different cell types within the tissue (acquired at ID16, ESRF; scale bar, 10 μm).

Figure 2 shows XPCT of a naïve mouse brain region where the VN (Figures 2A–C) and NN (Figures 2D–F) are virtually extracted independently by an image post-processing, i.e., segmentation process (see section “Materials and Methods”). Figure 2B presents a detail of the vascular network, while a zoomed image of the small capillary network obtained through holo-nano-XPCT is shown in Figure 2C. In the latter image, holo-nano-XPCT distinguishes two different types of cells: the black-appearing cells at the capillary walls, which are compatible with endothelial cells, and cells surrounding the capillary, which display a white cytoplasm and a dark nucleus. Of note, nano-XPCT and holo-nano-XPCT are presently the unique techniques able to image the 3D capillary network. Figure 2E shows the hippocampal NN, while Figure 2F displays a magnification of the tissue where a cell with typical pyramidal neuron morphology with dark cytoplasm, and surrounded by round cells, is clearly defined.

### EAE

Multiple sclerosis is a neurodegenerative autoimmune disease of the CNS associated with neuroinflammation, demyelination, axonal damage, and neuronal loss. The use of an appropriate animal model, such as EAE induced by MOG peptide, a chronic neurological disease with progressive caudo-rostral paralysis associated with demyelination and axonal loss (Mendel et al., 1995), facilitates the study of the disease mechanisms. In a previous XPCT study (Cedola et al., 2017), we demonstrated the presence of EAE-mediated vascular alterations down to the capillary network, and shed light on how the disease affects the tissues and on how treatment with MSC reverts the damage to some extent.

**FIGURE 3:**
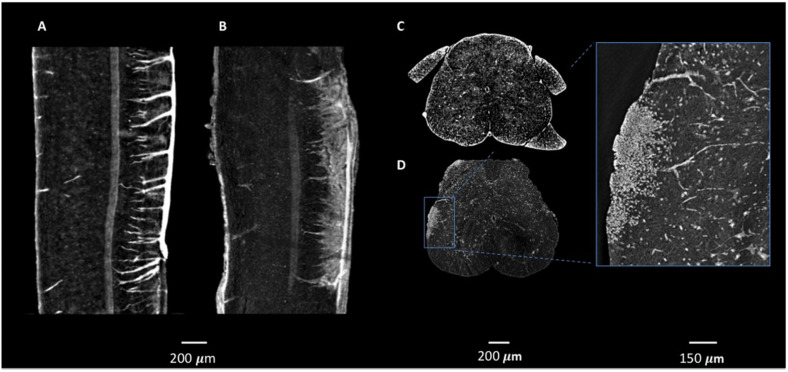
Micro-XPCT imaging of BBB leakage and lesion in lumbar spinal cord of EAE-affected mouse. **(A–D)** XPCT images showing the sagittal **(A,B),** and axial **(C,D)** views of the lumbar spinal cord in a naïve mouse **(A,C),** and an EAE-affected mouse at disease onset **(B,D)**, where vessels appear surrounded by numerous “clouds” of extravasated material **(B)** reflecting the intense BBB dysfunction in the EAE-affected mouse, and the accumulation of cells around the leaky vessels **(D)** is commensurate with the classical EAE lesion. Images were obtained as MAX of volumes acquired at ID17, ESRF **(A–C)** and at ANATOMIX, Soleil **(D)**. (**A–B**: MAX over 50 μm; **C,D**: MAX over 10 μm).

**FIGURE 4:**
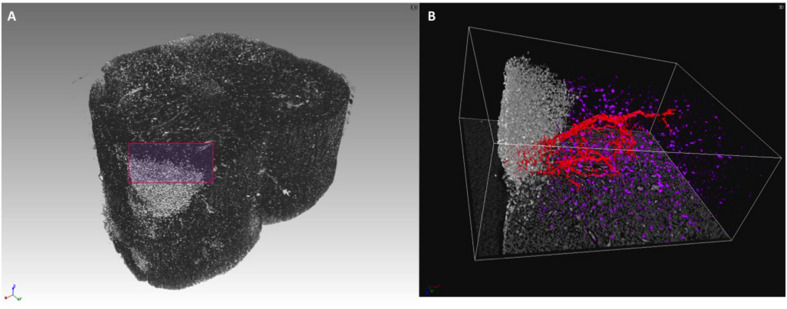
3D rendering of lumbar spinal cord lesion in EAE-affected mouse. **(A)** 3D rendering of a lumbar spinal cord volume (about 1-mm length) of EAE-affected mouse imaged with micro-XPCT at ANATOMIX, Soleil. **(B)** Detail of a lesion from **(A)** rendered and segmented in 3D. Images were obtained with VGstudioMax.

**FIGURE 5:**
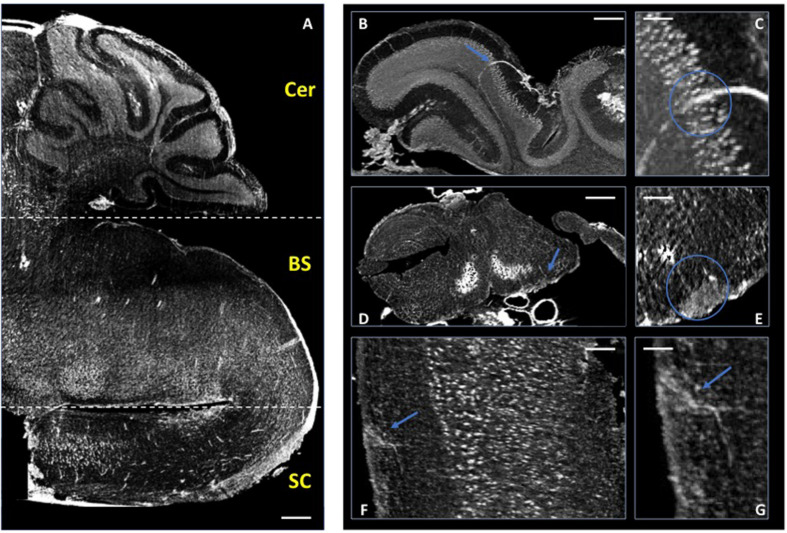
Micro-XPCT imaging of EAE-affected mouse CNS. **(A)** Micro-XPCT image showing a sagittal view of the cerebellum (Cer), brain stem (BS), and cervical section of the spinal cord (SC) of an EAE-affected mouse at disease onset (MAX over 150 μm; scale bar, 300 μm). **(B,C)** Micro-XPCT image of a detail of vessel in the cerebellum is shown in **(B)**, with a zoomed image of the same region shown in **(C)** (MAX over 130 μm; scale bar, 100 and 50 mm, respectively). **(D,E)** Inflammatory lesion with BBB leakage in the brain stem **(D)** zoomed in **(E)** (MAX over 65 μm; scale bar, 350 and 100 μm, respectively), and in the cervical portion of spinal cord **(F)**, zoomed in **(G)** (MAX over 30 μm; scale bar, 90 and 50 μm, respectively). Blue arrows indicate lesions, with blue circles encompassing the vessels and extravasated material. Images were acquired at ANATOMIX, Soleil.

In these previous studies of murine CNS tissue, we had perfused the mice with saline and heparin to remove the blood, or with MICROFIL^®^, a compound that fills vessels and enhances the opacity of the microvascular network (Fratini et al., 2015; Bukreeva et al., 2017; Cedola et al., 2017). In the present study, we made the serendipitous discovery that blood itself makes for a better contrast agent, with less manipulations. Indeed, we had planned to use gadolinium as a contrast agent to enhance the visibility of the vessels. We injected gadolinium intravenously, as it is used in human disease, and compared the images of the tissues with those from mice we had injected with PBS. Unexpectedly, we found that the XPCT images of brain samples from mice injected with gadolinium (data not shown) did not show better contrast than those from mice injected with PBS, where the presence of iron in the blood permits the visualization of the vessels. Accordingly, we have subsequently assessed the vascular networks in non-perfused mice that had not received contrast agent. More importantly for our study, the presence of iron in the blood also permits the visualization of extravasated material, as a result of BBB dysfunction. In 2D analysis, extravasation from CNS blood vessels, in particular capillaries, is detected most appropriately in mice upon intravenous injection of immunofluorescent material of high molecular weight, such as FITC-labeled dextran 70, prior to sacrifice, and appears in optical fluorescence microscopy as “clouds” of material diffusing in the parenchyma (Ferrara et al., 2016). Similar “clouds” can be seen in the XPCT images of brain and spinal cord from EAE-affected mice (Figures 3–5). EAE is an experimental disease where neurological impairment proceeds as a caudo-rostral ascending paralysis that is associated with inflammation and accumulating vasogenic edema, followed by demyelination and axonal damage. A dysfunctional blood-CNS barrier is first observed in the lumbar spinal cord, spreading to the upper spinal cord regions to reach the cerebellum. Figure 3 shows XPCT images of the lumbar spinal cord of a naïve mouse (Figures 3A,C) and of an EAE-affected mouse at the onset of the disease (day 11 after immunization; Figures 3B,D). In both sagittal (Figures 3A,B) and axial (Figures 3C,D) views, 3D analysis with XPCT shows clear alterations in this region. Thus, while we see well-defined vessels arising from the longitudinal spinal artery in the naïve mouse lumbar spinal cord (Figure 3A), these appear very fuzzy and surrounded by numerous “clouds” in the lumbar spinal cord of the EAE-affected mouse (Figure 3B), reflecting the intense BBB dysfunction at this stage of the disease. The 3D axial view of the lumbar spinal cord of the EAE-affected mouse shows very clearly a large accumulation of cells close to vessels (Figure 3D), which would be typical of an EAE lesion with infiltrating inflammatory cells, and is never observed in naïve mouse spinal cord (Figure 3C). In Figure 4, we see a 3D-rendering of spinal cord volume where localized extravasated material (bright gray) is clearly visible (panel A); in panel B, the 3D-rendering of the lesion in Figure 4A, emphasizes the surroundings of the vessel (red) by small cells (white) that differ from morphologically neuron-like cells (purple). Clouds reflecting BBB dysfunction together with accumulation of cells are also observed in the volume from the cervical region of the spinal cord (SC) up to the brain stem (BS), and cerebellum (Cer) of the same EAE-affected mouse, albeit at apparently much reduced frequencies (Figure 5A), as would be expected. Details of these three CNS regions are reported in Figures 5B,C (Cer), 5D,E (BS), and 5F,G (SC). The blue arrows (Figures 5B,D,F, and G) and circles indicate the areas where an extravasation is evident and appears as a white “cloud” close to an interruption or a thinner tract of the vessel. A clear gathering of small bright spots around the “clouds” (see in particular Figure 5E) suggests the presence of inflammatory cells in these areas, that could be compatible with infiltrating inflammatory T cells and macrophages, and/or microgliosis, which have all been extensively described in EAE (Constantinescu et al., 2011).

The scope of the present report was to provide an overview of the unique possibilities of XPCT and of the quality of the 3D images obtained that has allowed us to understand the extent of the BBB dysfunction in EAE. Rather than providing biological statistics, we have therefore presented a qualitative study with one mouse sample per condition. However, the high quality of our images has enabled us to quantify the number of BBB alterations at different time points throughout the disease, which have indicated an increased trend as function of the disease stage (manuscript in preparation).

### Alzheimer’s Disease

**FIGURE 6:**
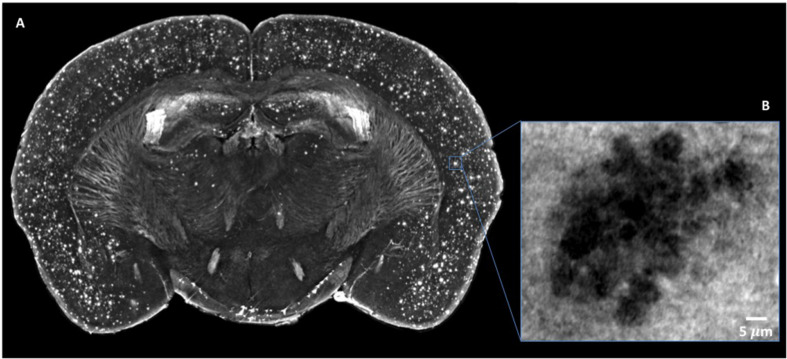
Micro-XPCT reveals the widespread distribution of Aβ plaques in cortex of 22-month-old AD mouse. **(A)** Maximum z-projection (over 100 μm) of micro-XPCT of AD mouse brain clearly shows dramatic presence of Aβ deposits localized all over the cortex. **(B)** shows an holo-nano-XPCT magnification of a Aβ plaque in an AD mouse cortex (the gray levels are inverted in the **(A)** and **(B)**, see the text). Images were acquired at ID16, ESRF.

**FIGURE 7:**
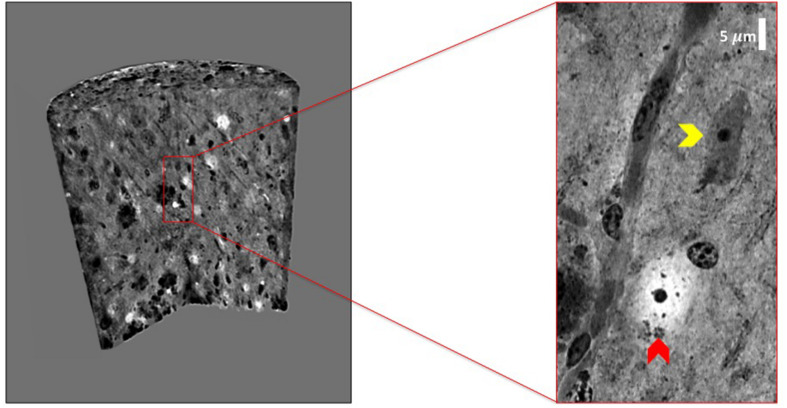
Holo-nano-XPCT enables the visualization of neuropathological findings in AD mouse brain tissue at capillary and cell levels. The volume of AD mouse brain tissue was rendered with 3D Viewer ImageJ plug-in. The inset shows a detail of a capillary revealing the presence of intralumen Aβ deposits. As can be seen in the inset, the high sensitivity of XPCT imaging allows the clear detection of cells with very different cytoplasm density, which might reflect the known presence of degenerated neurons (low cytoplasm density; red arrow), together with seemingly healthy neurons (high cytoplasm density; yellow arrow). Images were acquired at ID16 and ESRF.

We have exploited XPCT to study the APP/PS1dE9 mouse model of AD (Balducci and Forloni, 2011), a progressive neurodegenerative disorder associated with aberrant production of Aβ depositing in the brain as extracellular plaques, especially in the cortical and the hippocampal areas (Huang and Mucke, 2012). APP/PS1dE9 mice develop crucial AD signatures including extracellular and intravascular plaque deposition, cognitive impairment, and neuroinflammation. Figures 6A,B shows an XPCT image of an APP/PS1dE9 mouse brain section. Here, a large number of plaques appear as small bright spots (highly dense tissue) of a few tens of microns in size in both the cortex and the hippocampus. We used holo-nano-XPCT to achieve single plaque details, such as the presence of neurites – appearing as black dense spots – inside and around the plaque corona (Figure 6B). In AD, the deposition of Aβ inside the vessels determines their lumen reduction or even occlusions, as well as wall breakages, culminating in a reduced cerebral blood flow (Klohs, 2019). Holo-nano-XPCT can reveal fine details from a 3D volume (Figure 7, left panel) to capillary and cell levels. As can be seen in the inset of Figure 7 (right panel), holo-nano-XPCT has the ability to reveal the presence of intralumen deposits completely occluding the capillary. In this inset, holo-nano-XPCT also highlights the difference between healthy neurons featured by a dark-appearing dense cytoplasm (yellow arrow) and the degenerated ones with a low-density, white cytoplasm (red arrow) (Kuljis et al., 1997).

**FIGURE 8:**
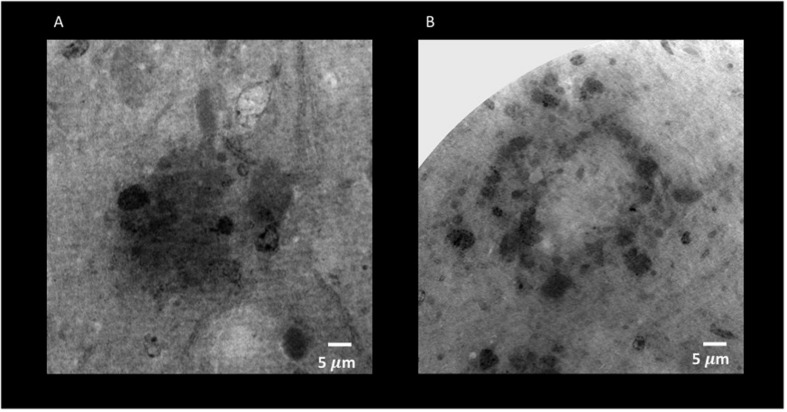
Holo-Nano-XPCT allows the visualization of fine details inside equal-sized areas of AD mouse cortex, revealing destructured plaques in AD mouse treated with MSC-CS. The high resolution of this powerful technique highlights differences in the structure of Aβ plaques in untreated **(A)** and MSC-CS-treated **(B)** mouse cortex. Images were acquired at ID16 and ESRF.

**FIGURE 9:**
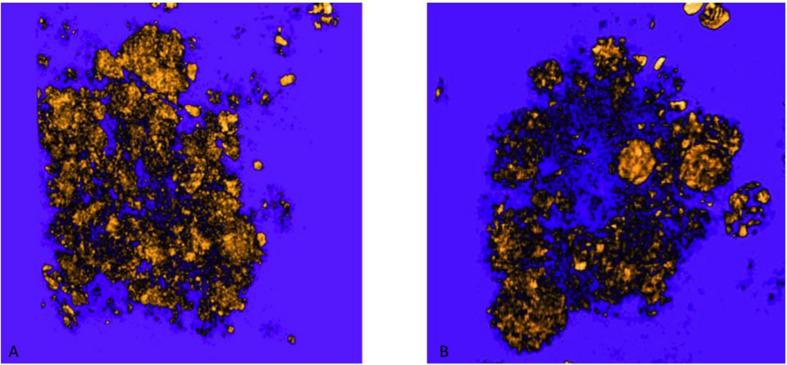
3D rendering of Aβ plaques in untreated and MSC-CS treated mouse cortex. **(A)** and **(B)** correspond to panels **(A,B)** of Figure 8, respectively. Images were obtained with VGstudioMax.

**FIGURE 10:**
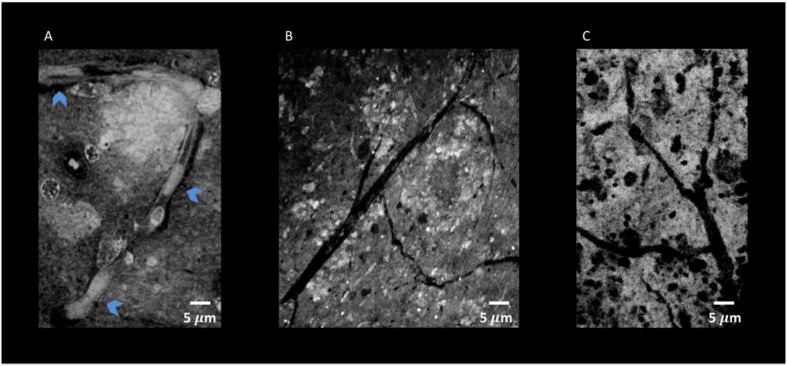
Nano-XPCT and holo-nano-XPCT permit the visual dissection of vessels in MSC-CS-treated and untreated AD mouse cortex. **(A)** Nano-XPCT image showing the almost complete occlusion of vessel lumen by Aβ deposits in vehicle-treated AD mouse brain. The image was acquired at ID16, ESRF. **(B)** Holo-nano-XPCT image showing cleaner vessels in the brain of AD mouse treated with MSC-CS. **(C)** Nano-XPCT image of vessels in a healthy wild-type mouse brain. Images were acquired at TOMCAT and SLS. All the images are the sum of MAX and MIN over 20 μm. Gray levels have been made consistent: white represents highest-density structures, black corresponds to less dense features.

Through holo-nano- and nano-XPCT, we could also investigate structural changes in APP/PS1dE9 mouse brain at an advanced stage of the disease, treated either with vehicle or with a conditioned secretome collected from mouse bone marrow MSC. These MSC had been primed with APP/PS1dE9 mouse brain homogenate, in order to mimic a typical AD microenvironment, which licenses the cells to assume a neuroreparative/immunomodulatory phenotype reflected in their secretome (MSC-CS). In a very recent study, we demonstrated the enormous therapeutic potential of MSC-CS intranasally administered in 22-month-old APP/PS1dE9 mice at a very advanced disease stage. MSC-CS reduced brain amyloidosis, neuroinflammation and hippocampal atrophy, increased neuronal density in both the cortex and the hippocampus, and improved mouse longevity considerably (Santamaria et al., 2020). By applying holo-nano- and nano-XPCT, we could achieve the resolution to visualize plaque and vessel details, which point to a significant effect of the treatment on the microenvironment whereby the treatment is associated with destructured plaques (Figures 8, 9 and Supplementary Movies 1 and Supplementary Movies 2). Thus, while in the brain from the vehicle-treated AD mouse we detected highly dense Aβ plaques surrounded by blood vessels almost completely occluded by the presence of Aβ deposits in their lumen (Figure 10A), in the AD mouse treated with MSC-CS cleaner vessels could be observed in close proximity to less dense neuritic plaques (Figure 10B). This latter vessel condition was highly comparable to what can be observed in the healthy WT mouse (Figure 10C).

## Discussion

To investigate crucial neuropathological signatures and therapeutic efficacy at fine level would require direct imaging of the whole brain in a 3D fashion, allowing for simultaneous analyses of (i) brain alterations in various cell populations (neuronal loss, gliosis, peripheral infiltrates); (ii) structural changes in terms of cell density and organization (brain atrophy); (iii) modifications in vascular networks and integrity (cerebral amyloid angiopathy, BBB modifications). This would be far more informative than the standard histological examination of isolated slices of brain tissue having undergone blood removal, freezing at extremely low temperature, and manipulation with aggressive fixatives and various detergents. Indeed, the conventional indirect immunohistochemical techniques require chemical tissue manipulations to add fluorescence/chromogenic reporters. MRI helps to circumvent these problems, but remains limited by a spatial resolution of a few mm or several tens of microns in preclinical studies. These limitations are overcome by X-ray tomography, which, however, has a poor performance in imaging soft tissues. This is why XPCT, which does not require processing of the tissue and makes possible multi-scale 3D imaging of NN and VN ranging from cells through to brain as a whole, can offer a valuable alternative. XPCT can reach up to a 1000-fold gain in contrast resolution with respect to conventional tomography and is therefore particularly useful for low-absorbing bio-medical samples. In particular, due to its high resolution and large FOV, XPCT is rapidly gaining importance in the investigation of neurodegenerative diseases, such as AD and MS. The use of animal models to mimic neurodegenerative disorders allows the investigation of neuropathogenic mechanisms and the monitoring of disease progression and therapeutic efficacy.

To the best of our knowledge, this is the first study that has been able to show the extent of BBB damage in EAE at the level of the single vessel/capillary. BBB dysfunction plays a paramount role in the development of EAE and MS and has been extensively studied. Until MRI and the development of means to apply it in small animals, BBB dysfunction was, and still is, generally studied at 2-dimensional level with biochemical, histological, and immunohistochemical methods (Kassner and Merali, 2015), that have included the assessment of extravasated albumin and/or IgG in CNS through immunoblotting (Kerlero de Rosbo et al., 1985), proteomic analysis (Han et al., 2008; Farias et al., 2012; Rosenling et al., 2012), and Evan’s blue or radiological labeling (LeVine, 2016). More recently, other injected tracers have included horseradish peroxidase that can be visualized through immunohistochemistry upon application of its substrate to 2D slices (Muller et al., 2005) and fluorescein isothiocyanate-labeled high molecular weight (70 kDa) dextran visualized by fluorescent microscopy of tissue slices (Ferrara et al., 2016). All these techniques require processing of the tissues to a greater or lesser extent, and do not allow the concomitant assessment of entire VN or NN. Multi-photon and two-photon microscopy is an *in vivo* method of assessing BBB disruption (Abulrob et al., 2008); however, albeit with high spatial and temporal resolution, it only permits the visualization of surface microvasculature because of poor tissue penetration (no more than 1-mm depth) and light scattering; it would therefore be inadequate to assess lesions in EAE in general, in particular perivascular and cortical lesions (Constantinescu et al., 2011). MRI has been used for 3D assessment of BBB dysfunction in EAE (Rausch et al., 2003; Nathoo et al., 2014), which enabled the demonstration of gadolinium-enhanced areas indicative of leakage across the BBB. However, unlike XPCT, the resolution of MRI does not reach the singular vessel or cell levels. Thus, to clearly show cell infiltration and accumulation of cells at BBB damage areas necessitates the adjunct use of 2D histology/immunohistochemistry (Kassner and Merali, 2015) with difficulty in matching the exact co-location of the lesion with both techniques. The injection of iron oxide-based nanoparticles that label macrophages in the CNS (Rausch et al., 2003) has also been used in conjunction with MRI, albeit with caution as such nanoparticles might not be easily distinguished by MRI from iron naturally deposited due to the disease process. In contrast, XPCT is able to visualize the fine location of the BBB leakage, together with its consequences, that is infiltrating and resident cell accumulation, in a single assessment on tissue that has not undergone extensive denaturation or processing. We believe that, as the technique improves, we will be able to track BBB lesions *in vivo* to thereby monitor disease progression and assess the efficacy of therapeutic approaches.

While our studies were the first to apply XPCT to evaluate VN and NN in EAE (Fratini et al., 2015), XPCT was used to assess the AD mouse model, showing the presence of plaques (Astolfo et al., 2016). In the present qualitative study, XPCT enabled us to demonstrate capillary occlusions and damages, close associations between plaques and damaged vessels, as well as dramatic changes induced by MSC-CS treatment in AD mice. Indeed, we could evidence clearing of Aβ deposits inside the vessels, as well as impressive changes in plaque structure and density.

The use of only one animal per group could be taken as a limitation of our study. However, rather than obtaining statistical data for a quantitative study, our aim was to emphasize the innovative aspect of XPCT in detecting pathological differences even at cellular level, in preclinical models of neurodegenerative diseases.

Our results put in evidence the essential use of XPCT as a cutting-edge technique able to provide further depth in the imaging of the damaged brain, without lengthy and/or destructive processing. While the present work does not provide statistically significant biological results, it shows how biological problems should be tackled to confirm and extend the observations obtained with standard techniques.

## Data Availability Statement

All datasets presented in this study are included in the article/Supplementary Material.

## Ethics Statement

The animal study was reviewed and approved by Ethical Committee for Animal Experimentation of the University of Genoa (Prot. 319) and the Istituto di Ricerche Farmacologiche Mario Negri IRCCS.

## Author Contributions

AC, CB, and NKdeR conceived and designed the experiments and participated to the discussion of the results and wrote the manuscript. MF, FP, NP, LM, GP, AS, and AC performed the experiments and contributed to the data analysis. IB, AC, MF, FP, NP, LM, GP, AS, IB, and GG have contributed to the discussion of the results and to the final revision of the manuscript. MC performed the data analysis. All the authors contributed to the final writing of the manuscript.

## Conflict of Interest

The authors declare that the research was conducted in the absence of any commercial or financial relationships that could be construed as a potential conflict of interest. The reviewer SG declared a shared affiliation with several of the authors, AU and NKdeR, to the handling editor at the time of review.

## Footnotes

1 https://imagej.net/Fiji
