# Corrigendum: X-ray Phase Contrast Tomography Serves Preclinical Investigation of Neurodegenerative Diseases

**DOI:** 10.3389/fnins.2021.657368

**Published:** 2021-02-17

**Authors:** Francesca Palermo, Nicola Pieroni, Laura Maugeri, Ginevra Begani Provinciali, Alessia Sanna, Inna Bukreeva, Lorenzo Massimi, Maura Catalano, Margie P. Olbinado, Michela Fratini, Antonio Uccelli, Giuseppe Gigli, Nicole Kerlero de Rosbo, Claudia Balducci, Alessia Cedola

**Affiliations:** ^1^TomaLab, Institute of Nanotechnology, CNR, Rome, Italy; ^2^Dipartimento di Fisica, Università della Calabria, Rende, Italy; ^3^Dipartimento di Morfogenesi e Ingegneria Tissutale, Sapienza Università di Roma, Rome, Italy; ^4^Swiss Light Source, Paul Scherrer Institut X-ray Tomography Group, Villigen, Switzerland; ^5^Department of Neurosciences, Rehabilitation, Ophthalmology and Maternal-Fetal Medicine (DINOGMI), University of Genoa, Genoa, Italy; ^6^Ospedale Policlinico San Martino IRCCS, Genoa, Italy; ^7^Institute of Nanotechnology, CNR, Università del Salento, Lecce, Italy; ^8^Istituto di Ricerche Farmacologiche Mario Negri IRCCS, Milan, Italy

**Keywords:** X-ray phase contrast tomography, preclinical disease models, Alzheimer's disease, multiple sclerosis, 3D imaging

In the original article, there were several errors, as detailed below.

**Author List, Affiliations, and Author Contributions**

Giuseppe Gigli was not included as an author in the published article. The corrected Author Contributions Statement appears below.

**Author Contributions**

AC, CB, and NKdeR conceived and designed the experiments and participated to the discussion of the results and wrote the manuscript. MF, FP, NP, LM, GP, AS, and AC performed the experiments and contributed to the data analysis. IB, AC, MF, FP, NP, LM, GP, AS, IB, and GG have contributed to the discussion of the results and to the final revision of the manuscript. MC performed the data analysis. All the authors contributed to the final writing of the manuscript.

In the original article, the affiliation for author Giuseppe Gigli was also not included. We have added the affiliation for Giuseppe Gigli as 7 - Institute of Nanotechnology, CNR, Università del Salento, Lecce, Italy.

Additionally, there was an error in the order of the authors in the author list of the original article. Inna Bukreeva should be the sixth author in the list. The original article has now been updated.

**Text Corrections**

In the original article, there was a mistake in the caption for Figure 1 as published. Figure 1A was adapted from Figure 3F of T. Zikmund et al 2018 JINST 13 C02039, but the reference to the paper was erroneously omitted. The corrected caption appears below.

Figure 1. XPCT is a powerful technique that provides high-contrast resolution without requirement for a contrast agent. The image shown puts together parts of naïve mouse brain with the left side assessed by standard X-ray micro-tomography with a mixture of 1% iodine and 90% methanol as contrast agent (A) as reported in Zikmund et al. (2018) JINST 13 C02039, while the right side was generated by XPCT (B). Small variations in density appear much more evident in XPCT imaging. Both images were obtained as z-projection of maximum intensity over 300 μm.

In addition, the full citation for Zikmund et al. (2018) JINST 13 C02039 will be added to the reference list of the original article.

The authors apologize for these errors and state that they do not change the scientific conclusions of the article in any way. The original article has been updated.
